# Apo, Zn^2+^-bound and Mn^2+^-bound structures reveal ligand-binding properties of SitA from the pathogen *Staphylococcus pseudintermedius*

**DOI:** 10.1042/BSR20140088

**Published:** 2014-11-24

**Authors:** Francesca Abate, Enrico Malito, Roberta Cozzi, Paola Lo Surdo, Domenico Maione, Matthew J. Bottomley

**Affiliations:** *Novartis Vaccines and Diagnostics srl, Via Fiorentina 1, 53100 Siena, Italy

**Keywords:** class III solute binding protein, divalent metal cation, manganese, staphylococcal disease, transport, zinc, ABC, ATP-binding cassette, CV, column volume, DSC, differential scanning calorimetry, FhuD2, ferric hydroxamate uptake D2, HIC, hydrophobic interaction chromatography, ITC, isothermal titration calorimetry, MR, molecular replacement, PDB, Protein Data Bank, rmsd, root mean square deviation, RP-HPLC, reverse-phase HPLC, SBP, solute-binding protein, SEC, size-exclusion chromatography, TEV, tobacco etch virus

## Abstract

The Gram-positive bacterium *Staphylococcus pseudintermedius* is a leading cause of canine bacterial pyoderma, resulting in worldwide morbidity in dogs. *S. pseudintermedius* also causes life-threatening human infections. Furthermore, methicillin-resistant *S. pseudintermedius* is emerging, resembling the human health threat of methicillin-resistant *Staphylococcus aureus*. Therefore it is increasingly important to characterize targets for intervention strategies to counteract *S. pseudintermedius* infections. Here we used biophysical methods, mutagenesis, and X-ray crystallography, to define the ligand-binding properties and structure of SitA, an *S. pseudintermedius* surface lipoprotein. SitA was strongly and specifically stabilized by Mn^2+^ and Zn^2+^ ions. Crystal structures of SitA complexed with Mn^2+^ and Zn^2+^ revealed a canonical class III solute-binding protein with the metal cation bound in a cavity between N- and C-terminal lobes. Unexpectedly, one crystal contained both apo- and holo-forms of SitA, revealing a large side-chain reorientation of His^64^, and associated structural differences accompanying ligand binding. Such conformational changes may regulate fruitful engagement of the cognate ABC (ATP-binding cassette) transporter system (SitBC) required for metal uptake. These results provide the first detailed characterization and mechanistic insights for a potential therapeutic target of the major canine pathogen *S. pseudintermedius*, and also shed light on homologous structures in related staphylococcal pathogens afflicting humans.

## INTRODUCTION

*Staphylococcus pseudintermedius* is a Gram-positive bacterium emerging as the most important pathogen causing canine pyoderma [1,2]. Moreover, *S. pseudintermedius* is associated with other canine diseases such as wound infections, urinary tract infections and otitis externa, thus cumulatively resulting in significant morbidity in dogs on a global scale. *S. pseudintermedius* was found to be one of the most common pathogens causing disease in thousands of small animal veterinary consultations [3]. Treatment of *S. pseudintermedius* in dogs is often ineffective, unless strong systemic antibacterials are used, thus risking an increase of antimicrobial-resistant bacteria in pets that can act as reservoirs for such pathogens, with a risk for transmission to humans [4,5]. Methicillin-resistant strains of *S. pseudintermedius* are emerging [1,2,6], frequently via acquisition of the *mecA* gene, thus resembling the MRSA (methicillin-resistant *Staphylococcus aureus*) infections currently increasing in humans and which caused over 18000 patient deaths prior to 2007 in the U.S.A. alone [7]. Although relatively rare, there are several reports of life-threatening *S. pseudintermedius* infections occurring in humans following dog bite wounds [8]. Consequently, the development of safe and effective therapeutics against *S. pseudintermedius* would have benefit in alleviating pathogen-induced disease and suffering, primarily in animals, and potentially also in humans [2].

Recently, the whole genomes have been sequenced for *S. pseudintermedius* strains ED99 [9] and HKU10-03 [10], providing important new resources for investigations of the molecular basis of canine bacterial pyoderma. Both strains exhibit a single circular chromosome with over 2400 predicted protein-coding sequences including numerous virulence factors, such as leucotoxins, exotoxins, adherence factors, superantigens, and other surface-exposed proteins, many of which may be interesting therapeutic targets [11,12]. For example, the cell-wall-associated proteins SpsD and SpsL are expressed by *S. pseudintermedius* during canine infection, are immunogenic and mediate binding to host extracellular matrix proteins, suggesting a role in disease pathogenesis [13].

Considering the similarities between *S. pseudintermedius* infections in dogs, and *S. aureus* infections in humans, increased knowledge of canine host–pathogen interactions involving *S. pseudintermedius* may potentially inform areas of human health and infectious disease, as well as veterinary medicine. Likewise, the wealth of information derived from extensive research into human infection by *S. aureus* may also promote an understanding of *S. pseudintermedius*. In particular, one set of surface-exposed proteins that has captured much attention in studies of staphylococci, and of pathogenic bacteria in general, is that of the SBPs (solute-binding proteins) [14,15]. SBPs act in concert with an integral membrane protein that mediates transmembrane solute transport, powered by an associated cytoplasmic ATP-binding protein, thus comprising the canonical bacterial ABC (ATP-binding cassette) transporter. A number of SBPs from both Gram-negative and Gram-positive bacteria have generated protective immunogenicity in animal models. Moreover, despite the possibility of steric hindrance due to the bacterial cell wall, anti-SBP antibodies can clear some bacterial infections and may inhibit bacterial growth by reducing their nutrient uptake. As such, ABC transporter components are generally considered interesting candidates for antibacterial vaccines or therapies [16].

In Gram-positive bacteria (including the staphylococci) SBPs are processed as extracellular surface lipoproteins, whereas in Gram-negative bacteria SBPs are localized in the periplasm and therefore were initially known as periplasmic binding proteins [14,15]. The SBPs typically share an overall architecture made of a ligand-binding groove located between two interfacing globular domains. Based on structure, the SBP family has been divided into three classes, depending largely on the linker(s) connecting the two globular domains [15]. The class I SBPs have three connecting segments between the two domains. In contrast, the class II SBPs have two interdomain crossovers, whereas the class III proteins only have one segment connecting the two domains [14,17]. Different SBPs function by binding to different solutes, including siderophores, carbohydrates, peptides, amino acids, and ions, and by shuttling these solute ligands to membrane-bound receptors for active transport into the bacterium; thus providing nutrients essential for survival, growth and virulence [14,15].

Some recently described examples of SBPs from *S. aureus* include FhuD2 (ferric hydroxamate uptake D2), which plays a role in staphylococcal dissemination [18] and indirectly binds Fe^3+^ via siderophores accommodated in a central pocket [19]; and MntC, an immunogenic surface lipoprotein [20] which directly binds Mn^2+^ ions [21]. Furthermore, the SBP structures of MtsA from *Streptococcus pyogenes*, and PsaA from *Streptococcus pneumoniae*, also reveal direct binding to Fe^2+^ and Zn^2+^, respectively, and their metal-binding ability is thought to have an essential role in virulence [22,23]. Interestingly, some SBPs can bind several different divalent metal cations, and competition by extracellular Zn^2+^ has been reported to inhibit bacterial uptake of Mn^2+^ [24], potentially reducing growth and virulence, due to the essential requirement for Mn^2+^ exhibited by many bacteria, including the staphylococci [25,26]. Indeed, both manganese and zinc are fundamental trace elements present in most organisms and are important for many biological processes, wherein these cations may act as cofactors of metallo-enzymes or as protein-stabilizing factors. Estimates of the zinc concentration in human plasma are at the low micromolar level, with reports ranging from 12 μM [27] to 2 μM bound zinc and as little as 30 nM for free zinc ions [28]. In a different study, it was estimated that human whole blood contains manganese at up to approximately 0.2 μM, most of which was found in erythrocytes [29]. Consequently, the potential competition between bacterial and host proteins for binding to metal ions will depend on the complex interplay of several factors, including the availability of free metals in the given circumstances, the abundance of chelating proteins, and their affinities for each metal ion; characteristics which are currently poorly characterized for the precise example occurring during *S. pseudintermedius* infections of dogs.

Despite this wealth of knowledge, and the sequencing of two *S. pseudintermedius* genomes, to date there is no detailed information available about the structure or function SBPs potentially present on the surface of *S. pseudintermedius*. Since such lipoproteins may play roles in virulence by scavenging essential solutes for active uptake from the host environment, we investigated the putative metal-binding protein, SitA, a potential therapeutic target of *S. pseudintermedius*. On a similar theme, recent studies of the close orthologue MntC from *S. aureus* showed that it can provide protective immunity in animal models of infection, suggesting that it has properties suitable for a candidate vaccine antigen [20].

Here we performed a detailed structural and functional study of SitA in order to fully characterize this *S. pseudintermedius* surface-exposed lipoprotein. We observed significant increases in the stability of SitA upon binding to divalent metal cations and took advantage of this to obtain crystals, which ultimately allowed determination of the structure of apo-, Zn^2+^- and Mn^2+^-bound forms of SitA. These structures, coupled with biophysical studies and site-directed mutagenesis, enabled precise identification of the key metal-binding determinants and provided insights into ligand specificity and the structural consequences of ligand binding that may govern ABC transporter engagement by SitA. The results discussed enhance our structural and functional understanding of the solute-binding proteins and ligand transport mechanisms in bacterial pathogens and in particular *S. pseudintermedius*.

## EXPERIMENTAL

### Molecular cloning and protein preparation

The SitA gene fragment encoding residues G23-Q306 was cloned by PCR, using the PIPE method [30], from *S. pseudintermedius* strain IV369-1041 (obtained from Quotient Bioresearch Ltd) (GenBank code CP002478). The SitA genes in the fully-sequenced HKU10-03 and ED99 genomes share 100% sequence identity with IV369-1041. The cloned fragment lacked the first 22 residues which encode a signal peptide for protein export and a lipobox cysteine residue. The fragment was inserted into a modified pET-15 vector (Novagen), enabling cytoplasmic expression of the SitA protein with an N-terminal 6-His tag followed by a cleavage site for the TEV (tobacco etch virus) protease. The PIPE method was also used to create two mutants by inserting the following point mutations in the SitA_23–306_ expression construct: H64A and E203A+D278A. All plasmids were verified by DNA sequencing. The residue numbering employed refers to the full-length SitA protein, Uniprot code F0P9H5.

The SitA expression constructs (wild-type or mutant clones) were transformed into chemically competent *Escherichia coli* BL21 (DE3) cells and the production of recombinant SitA was performed using the EnPresso Tablet Cultivation Set (BioSilta) growth system supplemented with 100 μg.ml^−1^ ampicillin. Bacteria were grown at 30°C for a total of 40 h and target protein production was induced by the addition of 1 mM IPTG (isopropyl β-D-thiogalactoside). Cells were harvested by centrifugation (6400 ***g***, 30 min, 4°C), resuspended in 50 mM sodium phosphate pH 8.0, 300 mM NaCl, and lysed by sonication (Qsonica Q700) for 5 min with cycles of 30 s sonication (40% amplitude) interspersed with 30 s on ice. Cell lysates were clarified by centrifugation at 36200 ***g*** for 30 min, and the supernatant was filtered using a 0.22 μm membrane (Corning filter system) prior to protein purification.

SitA was purified by affinity chromatography using an AKTA purifier 10 system (GE Healthcare). The filtered supernatant was loaded onto an Ni-NTA resin (10 ml column, GE Healthcare), and SitA was eluted using five steps of imidazole at 0, 25, 50, 75 and 250 mM concentration, at a flow rate of 5 ml.min^−1^. Fractions containing SitA were identified by a band migrating at ~35 kDa in SDS–PAGE analysis. The N-terminal 6-His tag was removed enzymatically by addition of a 6-His tagged solubility-enhanced TEV protease [31], with cleavage proceeding at room temperature overnight in buffer containing 50 mM sodium phosphate pH 8.0, 300 mM NaCl, 0.5 mM EDTA, 1 mM DTT (dithiothreitol). Subsequently, the sample was reloaded on the Ni-NTA resin to recapture the protease and free His tag, thus allowing elution in the column flow-through of SitA protein in the tagless form, which was used in all studies described herein. The SitA sample was concentrated and loaded onto a HiLoad Superdex 75 (26/60) preparative SEC (size-exclusion chromatography) column equilibrated in buffer containing 20 mM Tris–HCl pH 8.0, 150 mM NaCl, at a flow-rate of 1 ml.min^−1^. SitA protein was collected and 1 mM EDTA was added to remove divalent cations. The final yield of SitA obtained from 0.45 litre growth medium was over 200 mg (~11 mg protein per g wet biomass). The quality of the final sample was checked using 4–12% SDS–PAGE gradient gels in MES buffer. Sample purity was estimated to be >97%, for wild-type and mutant proteins, using RP-HPLC (reverse-phase HPLC) performed on a C4 column under standard conditions, essentially as described previously [32].

To separate the apo-form from metal-bound forms of SitA, an HIC (hydrophobic interaction chromatography) step was performed using a 5 ml HP butyl sepharose column (GE Healthcare) running at 1 ml·min^−1^. The column was equilibrated with ten CVs (column volumes) of buffer A (PBS containing 1.55 M sodium citrate, pH 7.2, 10 mM EDTA). The elution of the different forms of the protein was achieved with a linear gradient between buffer A and buffer B (PBS pH 7.2, 10 mM EDTA) in 60 CV. Two distinct peaks were eluted between 22 and 34% and between 51 and 86% of buffer B.

### DSC (differential scanning calorimetry)

The thermal stability of SitA proteins was assessed by DSC using a MicroCal VP-Capillary DSC instrument (GE Healthcare). SitA samples were prepared at protein concentration of 0.5 mg·ml^−1^ (~15 μM) in buffer containing 20 mM Tris–HCl, 150 mM NaCl, pH 7.5, with or without 2 mM MnCl_2_, ZnCl_2_, MgCl_2_ or CaCl_2_, with or without 10 mM EDTA. The DSC temperature scan ranged from 10 to 110°C, with a thermal ramping rate of 200°C·h^−1^ and a 4 s filter period. Data were analysed by subtraction of the reference data for a sample containing only buffer, using the Origin 7 software.

### CD

Far-UV CD spectra were recorded from 190 to 260 nm at 25°C using a Jasco-810 spectropolarimeter (Jasco). The cuvette chamber temperature was regulated using a PCB-1500 Peltier temperature system controller (Perkin-Elmer). SitA samples with or without 1 mM MnCl_2_ were prepared at 0.2 mg.ml^−1^ (~6 μM) in 5 mM KH_2_NaPO_4_ buffer, pH 7.2. A quartz cuvette with an optical path length of 1 mm was used. Spectra were acquired at 1 nm bandwidth, 0.5 s response time, 0.2 nm step size and 10 nm.min^−1^ scan speed. Each spectrum was calculated as the average of five accumulations. The spectra were corrected by subtracting the buffer baseline followed by application of a smoothing function in Spectra Manager v2.0 (Jasco).

### Crystallization, data collection and structure determination

For crystallization trials, purified SitA was concentrated to >150 mg.ml^−1^ using centrifugal concentration devices with 10 kDa molecular weight cut-off membranes (Amicon ultrafree, Millipore). Protein concentration was determined using Bradford assay (Bio-Rad, Protein Assay), and BSA as the reference. SitA in 20 mM Tris–HCl pH 8.0, 150 mM NaCl was prepared in three different ways for crystallographic screenings: (i) SitA apo protein, (ii) SitA+10 mM ZnCl_2_ and (iii) SitA+10 mM MnCl_2_. Drops of 200 nl SitA protein (with or without metal ions)+200 nl crystallization reservoir solution were dispensed using a Crystal Gryphon robot (Art Robbins Instruments). Metal ions were not added to the reservoir solution at any point. Crystals were grown in low-profile crystallization plates (Greiner) in a sitting-drop vapour diffusion format. Over 1500 crystallization conditions were screened at 20°C, with automatic imaging performed using a RockImager-182 (Formulatrix).

SitA crystals in presence of ZnCl_2_ or MnCl_2_ ions grew after incubation with a reservoir solution containing 30% (v/v) 1,1,1,3,3,3-hexafluoro-2-propanol (condition G3, MIDAS screen, Molecular Dimensions Ltd). Crystals were cryo-protected by addition of 20% (w/v) ethylene glycol prior to flash cooling in liquid nitrogen.

Diffraction data of SitA+Zn^2+^ were collected on beam line PXIII of the Swiss Light Source (SLS); whereas data of SitA+Mn^2+^ were collected on ID14-4 of the European Synchrotron Radiation Facility (ESRF). Diffraction data were processed using iMosflm [33] and scaled using AIMLESS in the CCP4 software suite [34]. Structure determination was performed using the Phaser Molecular Replacement software [35] with the structure of PsaA [PDB (Protein Data Bank) entry 1PSZ] as the search model. The 3D structures were refined using Phenix [36] and Buster [37], whereas model building was performed using Coot [38].

Crystals of SitA bound to Zn^2+^ or bound to Mn^2+^ belonged to space group *I* 2, with two chains of SitA in the asymmetric unit and with a solvent content of 39% (Matthews's coefficient 2 Å^3^.Da^−1^). Structural quality was assessed using Molprobity [39] and figures were prepared using PyMOL (http://www.pymol.org). Atomic coordinates of the SitA structures have been deposited in the PDB with entry codes 4OXR (Mn^2+^-bound dataset) and 4OXQ (apo/Zn^2+^-bound dataset). Pairwise structural comparisons of SitA with PsaA (Zn^2+^-bound form 1PSZ [22], Mn^2+^-bound form 3ZTT [24], and apo-form 3ZK7 [40]), TroA (Zn^2+^-bound form 3MFQ [41]), ZnuA (Zn^2+^-bound forms: 2OGW [42] and 2OSV [43]), MtsA (Fe^2+^-bound form: 3HH8 [23]) and MntC (Mn^2+^-bound form: 4K3V [21]), to obtain Cα rmsd (root mean square deviation) scores, were performed using SSM within Coot [38]. The rmsd values reported are the lowest rmsds obtained after making all possible pairwise comparisons of the different components of the respective asymmetric units, most of which contained two or four SBP molecules.

### ITC (isothermal titration calorimetry)

ITC measurements were performed using a MicroCal VP-ITC-200 instrument (GE Healthcare). ITC experiments were performed at 25°C. Both SitA and metal ion solutions were prepared in buffer containing 50 mM Hepes pH 7.5 and 50 mM NaCl. SitA protein concentrations were 10–20 μM. The metal ion solutions (MnCl_2_ and ZnCl_2_) were prepared at 100 μM concentration. Metal ions were injected at 5 min intervals, with 19, 37 or 73 injections of 2, 1 or 0.5 μl volumes, with injection times of 4, 2 or 1 s, respectively. Data were analysed using the Origin 7.0 software.

## RESULTS

### Apo-SitA is strongly stabilized by a subset of divalent metal cations

**Figure 1:**
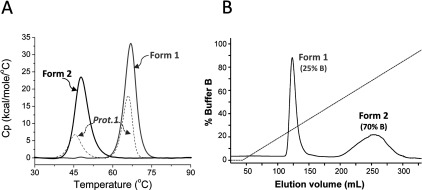
Differential scanning calorimetry profiles of different grades of SitA purifications (**A**) In DSC experiments a *T*_m_ value is given by the peak maximum of the scanned melting curve. The DSC profile of the first purified preparation of SitA (‘Prot.1’, dashed grey line), showed that the initial sample generated two peaks (*T*_m1_ 45.6°C and *T*_m2_ 66.0°C), consistent with two unfolding events, which could potentially be generated either by two different domains within one protein, or by two different forms of the same protein. The latter option was supported by HIC, from which two separate forms of SitA were obtained [Form-1 and Form-2, see panel (**B**)], each of which generated only one peak in DSC experiments (solid black and grey lines), *T*_m_ 47.7°C for Form-2, and *T*_m_ 66.9°C for Form-1. (**B**) The HIC elution profile of SitA, showing clear separation of two forms.

An expression construct for production of the SitA ectodomain (residues G23–Q306) was prepared from the *sitA* gene of a clinical isolate of *S. pseudintermedius*, strain IV369-1041 (see the *Experimental* section). Soluble recombinant SitA protein was produced in the cytoplasm of *E. coli* and was purified using standard chromatographic techniques, yielding a final protein purity level of >98%, as determined by RP-HPLC. Purity was estimated by measuring the area under the single sharp peak corresponding to SitA present in the chromatogram, with retention time approximately 3.7 min (Supplementary Figure S1). This first protein sample (termed ‘Prot.1’) was analysed by DSC, which revealed two distinct unfolding transitions with peaks at approximately 46 and 66°C (Figure 1A). This result suggested that the initially purified SitA might have been present in both apo- and holo-forms, potentially due to the partial capture of stabilizing ligands from the *E. coli* cytoplasm. Similar observations were described previously for the SitA homologue, MntC, from *S. aureus* [21].

Therefore an additional purification step using HIC was performed, enabling the separation of two distinct forms of SitA (Figure 1B), each of which produced a DSC profile with only a single peak, closely corresponding to one or other of the unfolding transitions originally displayed by Prot.1 (Figure 1A). Since ligand-binding often increases protein thermostability [44], we hypothesized that the peak with higher melting temperature (*T*_m_) was likely to correspond to a metal-bound holo-form of SitA, whereas the form of SitA with the lower *T*_m_ corresponded to apo-SitA.

**Figure 2:**
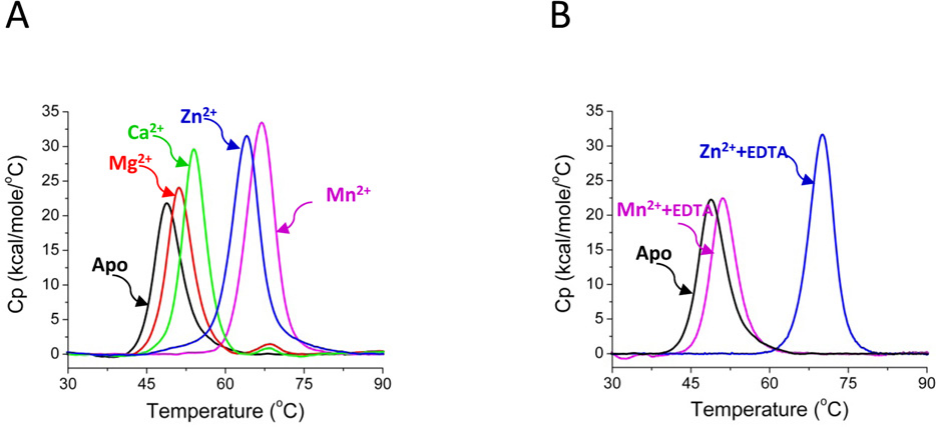
Different binding and reversibility of divalent metal cations with SitA (**A**) The purified recombinant apo-SitA protein displays a single peak, *T*_m_=48.7°C (black line), the lowest *T*_m_ measured in all these experiments. The DSC data for SitA+2 mM MnCl_2_ (magenta line, *T*_m_ 66.9°C) or SitA+2 mM ZnCl_2_ (blue line, *T*_m_ 64.1°C) displayed a single peak, corresponding to an unfolding event with high *T*_m_. The DSC data for SitA+2 mM CaCl_2_ (green line, *T*_m_ 53.9°C) or 2 mM MgCl_2_ (red line, 51.2°C) showed much smaller binding-induced increases in *T*_m_. (**B**) A strong chelating agent (EDTA, 10 mM) was added to SitA previously complexed with 2 mM Mn^2+^ or 2 mM Zn^2+^ and the DSC profiles were recorded. While the DSC profile of the Mn^2+^/EDTA-treated SitA sample (magenta line) revealed an unfolding transition which closely resembled that of metal-free apo-SitA (*T*_m_ 48.7°C, black line), the Zn^2+^/EDTA-treated SitA sample (blue line) revealed a *T*_m_ of 70.1°C, which approximately matched the profile of metal-bound SitA. (The reason for the minor difference in *T*_m_ between SitA+Zn^2+^ in the presence and absence of EDTA is currently unclear. Nevertheless, EDTA does not appear to stabilize SitA+Mn^2+^, revealing that SitA has a different propensity to bind the two metals under these conditions.)

The purified apo-SitA was subsequently examined by DSC for changes in thermostability upon the addition of various potential metal ligands. In the presence of 2 mM Mn^2+^ or Zn^2+^ ions, SitA displayed significantly increased *T*_m_ values: 66.9°C and 64.1°C, respectively (Figure 2A). In contrast, the stability of SitA was less dramatically increased upon addition of Mg^2+^ or Ca^2+^ ions, for which *T*_m_ values of only 51.2°C and 53.9°C, respectively, were obtained (Figure 2A). Despite significantly increasing the thermostability of SitA, metal cations did not induce notable changes in CD spectroscopy profiles (Supplementary Figure S2), suggesting that the secondary structure of SitA was not altered by ligand binding.

### SitA binds reversibly to manganese ions

It was demonstrated previously for the *S. pneumoniae* Mn^2+^ transporter PsaA that extracellular Zn^2+^ can competitively inhibit Mn^2+^ uptake into the bacterium and thereby negatively influence bacterial growth [45]. Moreover, in a recent report describing structural and biochemical studies, it was hypothesized that while the binding of PsaA to Mn^2+^ displayed an imperfect tetrahedral geometry (coordination number *n*=4) likely to facilitate Mn^2+^ ion release to the membrane-bound transporter, the same tetrahedral coordination was better suited for binding of Zn^2+^, which locked PsaA in a closed state such that Zn^2+^ binding was essentially irreversible [40]. To understand if SitA might display similarly selective metal-binding properties, apo-SitA was treated with 2 mM MnCl_2_ or ZnCl_2_ and, subsequently, was incubated with 10 mM EDTA. Indeed, in accordance with previous results for PsaA [40] and MntC [21], the resulting DSC profiles demonstrated that under these conditions in solution, the binding of SitA to Mn^2+^ was reversible by EDTA treatment, while binding to Zn^2+^ was not reversible (Figure 2B).

### The crystal structure of SitA with a bound manganese ion reveals a class III SBP fold

Since protein thermostability and, further, ligand-induced stabilization, have been shown to correlate with increased probability of protein crystallization [46,47], we sought to crystallize SitA in the presence of metal ions. A highly concentrated solution of SitA+10 mM MnCl_2_ was subjected to hundreds of crystallization trials, yielding reproducible crystals only in the presence of hexafluoroisopropanol.

**Figure 3:**
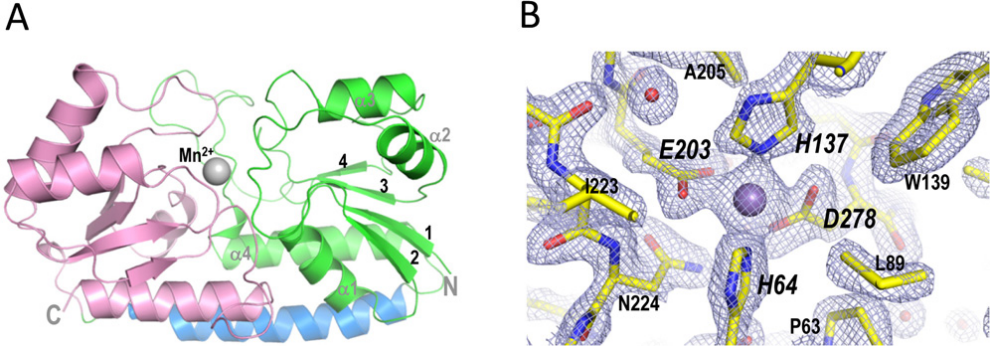
X-ray crystallographic structure of SitA bound to a Mn^2+^ ion (**A**) Cartoon representation of SitA showing the typical class III SBP fold: the N-terminal lobe (green) and C-terminal lobe (pink) are connected by a single long α-helix (blue). The Mn^2+^ ion, shown as a sphere, binds in a cavity between the two lobes. (**B**) Details in the ligand-binding pocket of Mn^2+^-bound SitA, with 2*F*o–*F*c electron density maps contoured at 2σ (shown as cyan mesh). Bond sticks are coloured by element, with carbon, oxygen, and nitrogen atoms coloured in yellow, red and blue, respectively. The Mn^2+^ ion is shown as a purple sphere; red spheres show water molecules. Key metal-binding residues are labelled in large italics, close neighbours are labelled in smaller non-italic font.

The structure of SitA bound to Mn^2+^ was determined by MR (molecular replacement). As the MR search model, we used the structure of PsaA (PDB code 1PSZ), which shares 48% sequence identity with SitA. The SitA+Mn^2+^ structure was refined at 2.0 Å resolution with the *R-*factor and free *R*-factor converging at 18.3 and 23.3%, respectively (see Table 1). The final refined model of SitA covered almost the entire protein, spanning residues K29–Q306 (Q306 is the actual C-terminus of SitA), and possessed excellent structure quality statistics (Figure 3A).

**Table 1:** Data collection and refinement statistics for two SitA structures Statistics for the highest-resolution shell are shown in parentheses.

	SitA+Mn^2+^	SitA+Zn^2+^
PDB ID	4OXR	4OXQ
*Data collection*		
Wavelength (Å)	0.9393	1.0
Beamline	ESRF, ID14	SLS, PXIII
Resolution range (Å)	46.45–2.0 (2.05–2.0)	51.42–2.61 (2.72–2.61)
Space group	I 1 2 1	I 1 2 1
Unit cell	80.58, 57.49, 112.3, β=101.95	81.66, 58.17, 112.3, β=101.8
Total reflections	114991 (8534)	43067 (5332)
Unique reflections	33582 (2426)	15736 (1922)
Multiplicity	3.4 (3.5)	2.7 (2.8)
Completeness (%)	98.2 (96.9)	98.9 (95.6)
Mean *I*/sigma(*I*)	11.4 (4.7)	3.7 (1.8)
Wilson B-factor	16.6943.2	
*R*_sym_*	11.6 (61.4)	16.8 (44.2)
*R*_meas_**	16.4 (86.9)	23.4 (61.1)
*Refinement*		
Resolution range (Å)	39.4–2.0	51.42–2.61
*R*_work_†	18.3	17.5
*R*_free_††	23.3	24.7
*Number of atoms*		
Macromolecules	4444	4420
Ligands	2	1
Water	181	186
Protein residues	29–306 (chains A and B)	29–306 (chain A), 29–125/129–306 (B)
RMS(bonds)	0.010	0.010
RMS(angles)	1.453	1.180
*Ramachandran* (%)§		
Analysed	98	96
Favoured	97	96
Allowed	3	4
Outliers	0	0
Clashscore	3.7	1.2
*Average B-factor*		
Macromolecules	24.9 (chain A and B)	27.5 (chain A), 30.9 (chain B)
Ligands	7.4, 5.9 (Mn, chain C and D)	31.0 (Zn, chain C)
Solvent	21.9 (H_2_O)	20.8 (H_2_O)
H64 NE2	6.20, 5.85 (chain A and B)	25.16, 33.49 (chain A and B)
H137NE2	3.74, 9.90 (chain A and B)	13.97, 18.53 (chain A and B)
E203OE1	5.43, 11.40 (chain A and B)	16.16, 19.77 (chain A and B)
E2030E1	6.32, 6.28 (chain A and B)	18.51, 21.78 (chain A and B)
D278 OD1	6.55, 10.66 (chain A and B)	13.21, 26.17 (chain A and B)
D278 OD2	8.43, 9.24 (chain A and B)	30.19, 30.51 (chain A and B)

*R_sym_=Σ*_hkl_* Σ*_i_* |*I_i_*(*hkl*)–<*I*(*hkl*)>|/Σ*_hkl_* Σ*_i_ I_i_*(*hkl*)

^**^R_meas_=redundancy-independent (multiplicity-weighted) *R*_merge_ as reported from AIMLESS [58].

†*R*_work_=Σ||*F*_(obs)_|−|*F*_(calc)_||/Σ|*F*_(obs)_|

††*R*_free_=as for *R*_work_, calculated for 5.0% of the total reflections, chosen at random and omitted from refinement.

**^§^**Figures from Molprobity [39].

The protein crystals obtained in the presence of Mn^2+^ contained two chains of SitA per asymmetric unit, arranged in a ‘face-to-face’ manner (Supplementary Figure S3). This dimeric organization is likely to be only a consequence of the very high protein concentration used for crystallization (>4 mM SitA). To allow a complementary non-crystallographic assessment, analytical SEC was performed with SitA at lower concentration (~100 μM), and yet still likely to be above the physiologically relevant concentration, revealing that SitA was monomeric in solution (theoretical monomeric molecular weight: 32 kDa, apparent molecular weight in SEC: 35 kDa). The SEC elution profile of SitA did not change upon addition of 10 mM MnCl_2_ or ZnCl_2_ (Supplementary Figure S4). Furthermore, analysis of the crystallographic dimer interface via the PISA (Proteins, Interfaces, Structures & Assemblies) software [48], did not detect any interfaces likely to result in the formation of stable quaternary complexes.

Structurally, the two chains of SitA bound to Mn^2+^ ions are essentially identical and can be superimposed with rmsd values of only 0.49 Å for all 278 aligned Cα atoms. For each chain, the overall monomeric structure is composed of two different lobes, or domains, connected by a long α-helix (residues A164–K188). The N-terminal domain (K29–T162) is composed of a single β-sheet of four short parallel β-strands partially surrounded by four α-helices. Similarly, the C-terminal domain (residues I192–Q306) is also composed of four short parallel β-strands and four α-helices (Figure 3A). Overall, the protein displays a bilobate bean-like structure, typical of the class III SBP family, with dimensions of ~60×43×43 Å. Electron density was clearly observed for almost the entire protein and for approximately 90 water molecules per chain. Moreover, electron density was clearly observed for a single Mn^2+^ ion per protein monomer, found in a pocket ~10 Å deep, located between the two lobes. The Mn^2+^ ion is chelated by nitrogen and oxygen side-chain atoms of His^64^, His^137^, Glu^203^ and Asp^278^ (Figure 3B).

A search of the PDB revealed several structures similar to SitA, all of which are class III SBPs, most notably including: *E. coli* ZnuA–rmsd 2.19 Å (for 240 aligned Cα atoms), *S. suis* TroA–rmsd 1.53 Å (250 Cα atoms), *S. pyogenes* MtsA–rmsd 1.12 Å (272 Cα atoms), *S. pneumoniae* PsaA–rmsd 1.03 Å (275 Cα atoms) and *S. aureus* MntC–rmsd 0.62 Å (276 Cα atoms). The folds of these proteins are well-conserved (for examples, see Supplementary Figure S5) and overall there are relatively few differences when comparing SitA with ligand-free or ligand-bound class III SBPs. Together with our CD spectroscopy data, these observations suggest that the ligands do not induce large changes in the secondary or tertiary structures of these proteins.

### SitA structures bound to Mn^2+^ or Zn^2+^ are highly similar overall

The DSC experiments described above demonstrated that the thermostability of SitA was most notably increased by Mn^2+^ and Zn^2+^ ions, and that upon addition of EDTA the binding in solution to Mn^2+^ was reversible, while binding to Zn^2+^ was not appreciably reversible. To understand whether the binding mechanisms were comparable, we sought to crystallize SitA in the presence of Zn^2+^ ions.

A SitA sample to which Zn^2+^ had been added produced crystals, again using hexafluoroisopropanol as the reservoir solution (to which metal ions had not been added), and the structure was determined by MR, using our refined structure of SitA bound to Mn^2+^ as the search model. The SitA+Zn^2+^ structure was refined at 2.6 Å resolution with the *R-*factor and free *R*-factor converging at 17.5 and 24.7%, respectively (see Table 1). As seen for the SitA+Mn^2+^ crystals, the SitA+Zn^2+^ crystals contained two monomers per asymmetric unit (despite the existence of SitA as a monomer in solution in the presence of ZnCl_2_). A pairwise comparison of the SitA monomer structures bound to Mn^2+^ or Zn^2+^ revealed that the two structures were almost identical overall (rmsd 0.41 Å). However, unexpectedly, the crystals obtained using the Zn^2+^-treated SitA revealed two slightly different monomer structures within the same asymmetric unit. Remarkably, while one chain was bound to Zn^2+^, the other monomer was present in the apo-form, lacking a bound metal ion. A detailed inspection of difference electron density maps around the Zn^2+^ binding pocket allowed modelling of one Zn^2+^ ion with 50% occupancy for one chain only (chain A). In contrast, for the other chain present in the asymmetric unit (chain B) the electron density in the binding pocket was compatible only with the presence of a water molecule, not a metal ion, supported also by the B-factor distribution of proximal atoms.

### SitA binds Mn^2+^ and Zn^2+^ with slightly different coordination geometry

**Figure 4:**
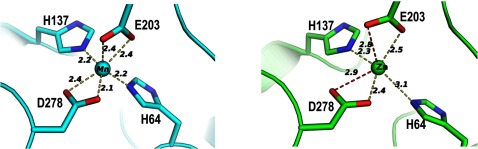
Atomic details of the SitA-metal cation interactions The cation-binding sites of Mn^2+^-bound SitA (cyan, left panel) and Zn^2+^-bound SitA (green, right panel) reveal that the binding mechanisms are very similar, with a small but notable difference in metal coordination observed for the His^64^ side chain. Notably, the side chain NE2 atom of His^64^ is 2.2 Å from the Mn^2+^ ion, but is shifted >1 Å further away (3.1 Å) from the Zn^2+^ ion. For each structure, the B-factors of the metal ions refined to values similar to those of the cognate protein atoms involved in metal chelation.

**Figure 5:**
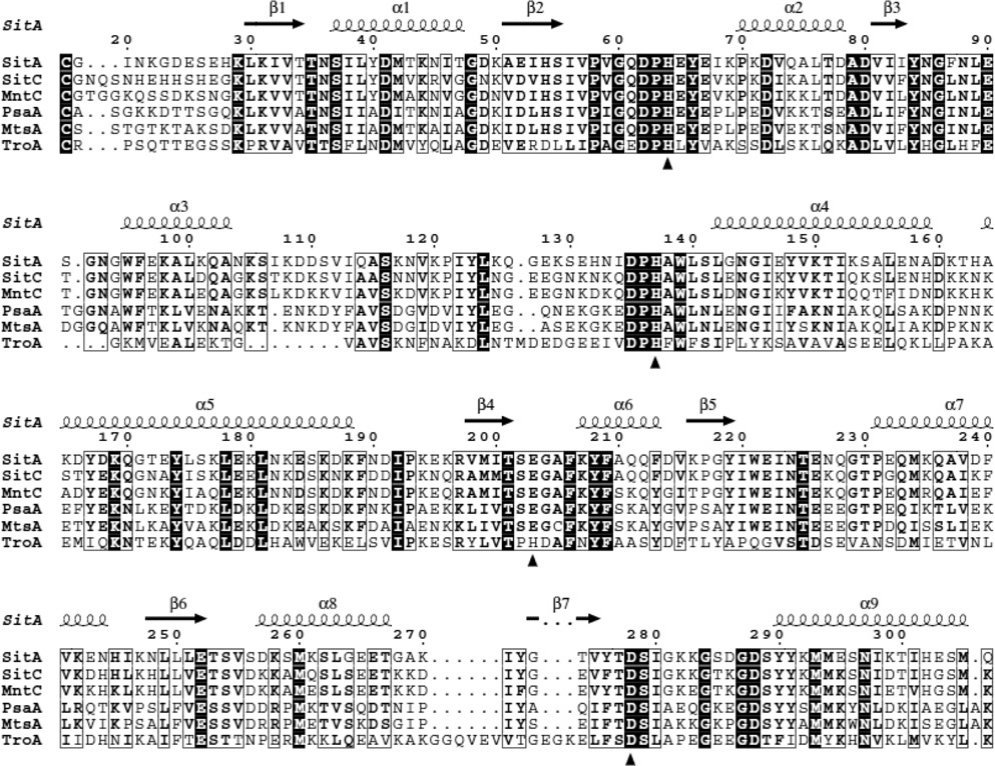
SitA orthologues show high sequence identity and conservation of metal-binding residues A multiple sequence alignment revealing the high degree of sequence identity (ID) between SitA and its orthologues: SitC in *S. epidermidis* (68% ID), MntC in *S. aureus* (65% ID), PsaA in *S. pneumoniae* (48% ID), MtsA in *S. pyogenes* (45% ID) and TroA in *S. suis* (27% ID). The alignments begin at the reactive Cys residue of the lipobox motif conserved in these Gram-positive lipoproteins. Residues are numbered according to full-length SitA, and are shaded black if fully conserved, or are boxed if partially conserved. Metal-chelating residues are indicated with black triangles. Secondary structure elements of SitA (derived from the Mn^2+^-bound structure) are shown above the sequences. Multiple sequence alignments and figures were prepared using the MAFFT algorithm [59] and ESPript [60].

The SitA structures revealed that the Mn^2+^ or Zn^2+^ ions were accommodated almost identically in the binding pocket. Electron density was clearly visible for all metal-chelating residues in the pocket, which is well-suited for divalent metal cations (Figure 3B). In both cases, the metal ion is coordinated by side-chain nitrogen and oxygen atoms of four tetrahedrally arranged residues: His^64^, His^137^, Glu^203^ and Asp^278^ (Figure 4). The metal-chelating residues are located in the lower regions of the binding pocket, and are contributed both by the N-terminal lobe (His^64^, His^137^) and the C-terminal lobe (Glu^203^, Asp^278^). For the Mn^2+^-bound structure, the positions of all four chelating residues, coupled with the bidentate nature of the Glu and Asp side chains, enables six coordinate bonds to the metal ion, with nearly perfect octahedral geometry and with protein–metal bond lengths of 2.1–2.4 Å (Figure 4A), as typically seen for Mn^2+^ [49,50]. In contrast, the binding pocket accommodates the Zn^2+^ ion in a less canonical manner, in particular because the nitrogen-to-Zn^2+^ distance involving His^64^ shows a 3.1 Å bond length, and the carboxylate oxygen-to-Zn^2+^ distances vary from 2.4 to 2.9 Å (Figure 4B); all of these are longer than the mean distances observed for the coordination of Zn^2+^ either in high-resolution protein structures [49] or in medium-resolution structures [50]. Although the medium resolution of the Zn^2+^-bound structure determined here (2.6 Å resolution, compared with 2.0 Å resolution for Mn^2+^) prompts a cautious interpretation of the precise protein–metal bond distances, the observations above suggest that the binding site is more typical of Mn^2+^-binding proteins rather than Zn^2+^-binding proteins, indicating transport of Mn^2+^ as a more likely biological function of SitA. This function is likely to be shared by other species, since all four metal-chelating residues are fully conserved in the orthologous SitA proteins of *S. aureus*, *S. epidermidis* and *S. pneumoniae* (Figure 5).

### The apo-SitA structure reveals a gating mechanism for entry to the metal-binding pocket

To gain insights into the putative ligand-induced mechanism resulting in fruitful engagement of the SitBC receptor by the metal-bound form of SitA, we sought to determine the structure of apo-SitA. Generally, it seems plausible to infer that the crystallization of apo-form SBPs is problematic, since relatively few apo-SBP structures are present in the PDB compared with the numerous holo-SBP structures available. Presumably this is because the ligand-enhanced stability promotes crystallization of the holo-forms, as discussed above, while the more dynamic ligand-free apo-forms are recalcitrant to crystallization. Indeed, we were unable to crystallize a purely apo-form of SitA. However, as described briefly above, using a Zn^2+^-treated sample, SitA crystals were obtained in which the asymmetric unit contained two polypeptide chains in different states, one of which presented SitA in the Zn^2+^-bound form (chain A), and the other of which presented SitA in the apo-form (chain B).

**Figure 6:**
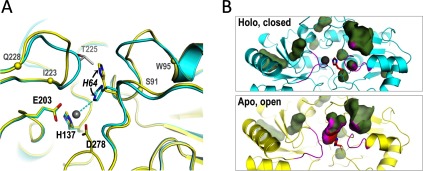
Differences proximal to the ligand-binding pocket in apo- and holo-SitA (**A**) Cartoon representations of the aligned structures of Zn^2+^-bound SitA (cyan, as in Figure 4) and apo-SitA (yellow) which were observed within the same asymmetric unit. The differences in the metal-binding site of apo- and holo-SitA are most notable in two loops S91–W95 and I223–Q228 (demarcated by Cα spheres on the apo- structure) surrounding the ligand-binding pocket and the outward position of His^64^ seen in the apo-structure, which is incompatible with the closed holo-structure, since it would clash with Thr^225^ (pale grey) in the holo-structure. Metal-chelating residues are labelled in black font, with the dynamic His^64^ in italics. (**B**) The conformational changes highlighted by the ribbon representations in panel (**A**) also lead to two rather different surface/cavity landscapes for the apo- and holo-forms of SitA. Here, blob-surfaces show internal cavities and/or surface-exposed tunnels/pockets. Most of the blobs are present in both states, showing that holo-SitA (upper panel) and apo-SitA (lower panel) surfaces/cavities are highly conserved. However, for apo-SitA, the additional large blob in the centre of the lower panel represents the tunnel leading from the apo-SitA surface down into the metal-free binding site. This tunnel disappears upon metal binding and closure, and hence the blob is absent in the upper panel. The flexible loops S91–W95 and I223–Q228 are coloured magenta, while sticks of His^64^ are coloured red and blue for Cα and nitrogen atoms. The side-chain contributions of the loops and of His^64^ to lining the walls of the cavities, are shown with magenta and red spots, respectively. All figures were made using Pymol v1.7, using surface ‘cavity’ mode for panel B.

A first comparison of the apo- and Zn^2+^-bound SitA structures revealed a very high degree of similarity, rmsd 0.39 Å (for 274 aligned Cα atoms). However, proximal to the metal binding site, in the loop spanning residues N224–N227, the Cα atom positions differed significantly (maximum displacement of 4.5 Å for E226), which appears to be the only notable change in the C-terminal lobe (Figure 6A). In addition, in the N-terminal lobe, also proximal to the metal, significant changes in loop S91-W95 were observed (maximum displacement of 1.9 Å for N93), and these changes are concomitant with small shifts of 0.5–0.7 Å in all Cα atoms in the immediately following α-helix (K98–A103), and in the spatially adjacent α-helix (E67–T77). Furthermore, in the apo-structure, there was increased flexibility (disorder) in the loop connecting K125–K129, for which electron density was not observed. Collectively, these small changes create local differences in the conformation and electrostatic surface profile of SitA in this region that SBPs typically use for binding to their cognate ABC transporters [51]. Most interestingly, the different local loop conformations render the binding pocket considerably more ‘open’ in the apo-structure, and ‘closed’ in the holo-structures. Concomitant with these local structural rearrangements, the side chain of residue His64 undergoes a remarkable reorientation by rotation of >100° around the Cα–Cβ bond, adopting a rotameric state that repositions the His^64^ NE2 atom >7 Å away from the potential metal-binding site, thus destroying the tetrahedral arrangement of amino acid side chains seen for metal coordination in the ‘closed’ holo-structure (Figure 6A).

Finally, a comparison of the apo- and holo-SitA structures showed that most of their pockets, surface-exposed tunnels and cavities are conserved. However, unlike the holo-form, the apo-form presents a large tunnel leading from the surface down into the metal binding site. This cavity is eliminated upon metal binding, due to ‘closure’ of the aforementioned loops (especially N224–N227) and repositioning of His^64^, in what resembles a ‘gating’ mechanism controlling entry of metal ions into the binding pocket (Figure 6A). A similar reorientation of an analogous histidine (His^67^) was also recently reported for PsaA, where the holo-form shows a ‘closed’ tetrahedral metal coordination geometry [22], whereas the apo-form reveals His^67^ in an outward-facing rotameric state due to a large rotation of the side chain [40]. These structural insights reveal the major differences between the apo- and holo-forms of SitA, which may underlie the ligand-dependent mechanism governing functional engagement of the SitBC receptor.

### Point mutations in the ligand-binding site abolish metal binding

**Figure 7:**
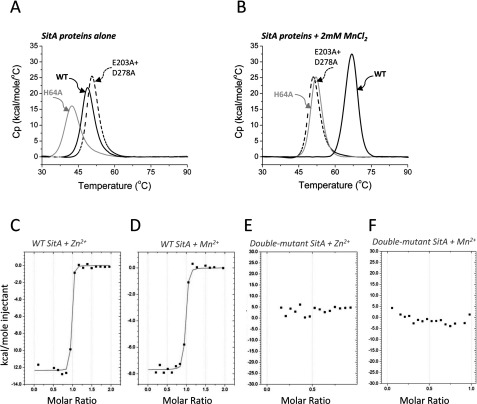
SitA point mutations abolish Mn^2+^-induced stabilization (**A**) DSC experiments performed in the absence of metal ions revealed only a single peak, with similarly low *T*_m_ values (40–50°C) for WT (solid black line, *T*_m_ 48.7°C) and the SitA mutants H64A (grey line, *T*_m_ 42.4) and E203A+D278A (dashed black line, *T*_m_ 50.6°C). (**B**) DSC experiments performed in the presence of 2 mM MnCl_2_ revealed that the H64A mutant (grey line, *T*_m_ 52.0°C) shows a small but notable increase in *T*_m_, whereas the double-mutant E203A+D278A (dashed black line, *T*_m_ 51.0°C) shows no change in *T*_m_. The WT protein (solid black line, *T*_m_ 66.9°C) exhibits the typical large increase in *T*_m_ in presence of Mn^2+^ ions. (**C**–**F**) ITC experiments to examine the interaction of wild-type and mutant SitA with Mn^2+^ and Zn^2+^ ions. Panels (**C**) and (**D**) show the high affinity interaction of Mn^2+^ and Zn^2+^ with wild-type SitA; panels (**E**) and (**F**) show data for the SitA E203+D278A double-mutant, which presented no detectable interaction with Zn^2+^ ions or Mn^2+^ ions.

To verify the importance of the metal-chelating residues in the SitA-binding pocket, single and double point mutants of SitA were prepared. The mutant proteins were successfully produced and purified as for wild-type SitA. The metal-binding properties of each mutant were examined by DSC. In the absence of metal ions, the double mutant E203A+D278A displayed a single peak corresponding to an unfolding event with a low *T*_m_ of approximately 45°C, equivalent to the low *T*_m_ observed for wild type apo-SitA (Figure 7A). In the absence of metal ions, the single mutant H64A showed a slightly reduced *T*_m_ (42°C), possibly because the substitution with Ala results in the loss of mildly stabilizing interactions occurring between the imidazole ring and residues in loops S91–W95 and N224–N227 which are proximal to the side chain of His^64^ in the apo-SitA structure (Figure 6A). Upon addition of 2 mM MnCl_2_, the wild-type SitA showed the usual large increase in thermostability (*T*_m_=67°C), while the mutants behaved differently. The H64A single mutant was stabilized by Mn^2+^ (*T*_m_ increased from 42 to 53°C)–though to a lesser extent than for wild-type SitA, and the E203A+D278A double mutant displayed essentially no change in *T*_m_ upon addition of Mn^2+^ (Figure 7B). Subsequently, we attempted to use ITC to determine the equilibrium dissociation constant (*K*_D_) for the binding of wild type SitA to Mn^2+^ and Zn^2+^ ions. The ITC data obtained indicated tight interactions of wild-type SitA with both metal ions. The binding curves relating the heats of injection to the molar ratio metal:SitA displayed sharp transitions, revealing that the interactions were apparently of high affinity, and were close to the upper limits that can be precisely detected by this technique [52]. Under these conditions, we estimated the K_D_ values to be in the low nanomolar range for both metal ions (Figures 7C and 7D). However, owing to the technical limitations discussed, we cannot exclude that the binding interaction of one or both metal ions was even tighter. More importantly, ITC measurements using the double-mutant E203A+D278A did not show any significant interaction with metal ions, confirming that this simultaneous pair of mutations completely abolished the Mn^2+^ and Zn^2+^-binding ability of SitA (Figures 7E and 7F). It was also recently shown that the corresponding residues in PsaA, namely E205 and especially D280, were essential for binding both Zn^2+^ and Mn^2+^ ions [40].

## DISCUSSION

Structural and biochemical studies of SBPs have provided many insights into the molecular mechanisms used by bacteria to obtain a wide range of nutritional solutes [14,15]. In particular, due to the necessity of metal ions in a wide range of important metabolic processes, modes of metal acquisition during staphylococcal infections have captured much attention. In this report, we have presented a series of investigations performed in order to fully characterize SitA, a surface-exposed lipoprotein from the animal and human pathogen, *S. pseudintermedius*. The structures presented herein are the first reports of protein structures from *S. pseudintermedius*. As discussed below, the structures, biophysical studies and site-directed mutagenesis, revealed the determinants of ligand binding in SitA, and unveiled subtle ligand-induced conformational changes that may underlie the molecular switch required for fruitful receptor engagement and ion transport.

Our studies were focused on the SitA protein from a clinical isolate of pathogenic *S. pseudintermedius*. Based on sequence analyses, SitA is annotated in the UniProt Knowledge-Base as a putative metal-binding protein. To explore this hypothesis experimentally, we performed CD and DSC experiments. Although the CD spectra did not reveal differences in the presence or absence of metals, the DSC experiments revealed a large stabilization of SitA in the presence of divalent metal cations. In particular, both Zn^2+^ and Mn^2+^ ions increased the *T*_m_ of SitA by ~20°C (Figure 2A). Furthermore, Mg^2+^ and Ca^2+^ also stabilized SitA, although to a much lesser extent, suggesting that there is distinct specificity for the transition metal ions. Indeed, following an analysis of thousands of metalloprotein structures in the PDB, it was reported previously that the alkali metals Mg^2+^ and Ca^2+^ are rarely coordinated by nitrogen (e.g. from histidine imidazole side chains, of which two are present in the SitA-binding pocket), but instead demonstrate a preference for the coordinating element to be oxygen, in particular deriving from an amino acid side-chain carboxylate group [49,50]. The strong intrinsic tendency of SitA to bind metal ions was also evident from the observation that recombinant SitA simply purified from *E. coli* lysates using EDTA-treated buffers displayed two peaks in DSC experiments–indicating that apo-SitA had partially gathered metal ions during production in the bacterial cytoplasm. The difficulty of precluding metal binding, even in the presence of strong chelating agents, has also been observed previously in studies of the proteins PsaA from *S. pneumoniae* [40] and MntC from *S. aureus* [21]. These observations support the hypothesis that SitA is involved in the capture of metal ions during the bacterial lifecycle. The amino acid sequence of the SitA protein studied herein is 100% conserved in the *S. pseudintermedius* genomes reported to date [9,10], suggesting that transition metal binding by SitA is important at least for these three strains of the pathogen. Interestingly, the binding of SitA to Mn^2+^ was found to be reversed by EDTA, while this chelating agent was not able to remove Zn^2+^ from SitA. Since EDTA is only one of many possible metal ion chelating agents, it is conceivable that alternative stronger metal ion binders might have induced different effects. Nevertheless, our observations suggest that Mn^2+^ may be more readily bound and then released compared to Zn^2+^, and thus is likely to be the more efficiently transported ligand. However, it cannot be excluded that the release and transport of Zn^2+^ might be promoted by conformational changes potentially occurring in SitA upon binding to the membrane-bound receptor.

To gain insights into the molecular mechanism of ligand binding, we sought to use X-ray crystallography to obtain the three-dimensional structure of SitA. Since protein crystallization is often facilitated by small-molecule ligands and high thermostability [46,47], we exploited the metal-induced thermostabilization reported above to generate co-crystals and initially determined the structure of Mn^2+^-bound SitA (Figure 3). The SitA structure revealed all the hallmarks of a class III SBP, presenting a bilobate structure of N- and C-terminal domains connected by an α-helix, with the Mn^2+^ ion harboured in a central ligand-binding pocket. The large interface between the two lobes, the characteristically long α-helix of 25 residues, and the ‘domain bridging’ effect of the Mn^2+^ ion coordinated by residues from both the N- and C-terminal lobes, collectively appear to confer rigidity to the overall protein, and presumably account for the high thermostability displayed by holo-SitA. The metal-induced stabilization presumably holds SitA in the optimal conformation required for effective receptor binding and subsequent ligand transport. Here, we used the previously reported coordinates of PsaA in order to determine experimentally the structure of Mn^2+^-bound SitA. PsaA is one of the class III SBPs produced by *S. pneumoniae* and is part of the well-characterized manganese ABC transporter system PsBCA [45]. Pairwise comparisons showed that SitA and PsaA share a sequence identity of 48% and exhibited very similar secondary structure compositions with 3D structures that can be superimposed with rmsd values of only 1 Å and revealing only minor structural differences in some loop regions (Supplementary Figure S5). This high degree of structural similarity strongly suggests a shared function in manganese binding and transport. Nevertheless, while Mn^2+^-bound SitA displays near-perfect octahedral binding geometry, this has not been equally observed in all Mn^2+^-binding SBPs, including PsaA, and MntC from the cyanobacterium *Synechocystis* [53]. Indeed, recently it was proposed that the imperfect metal coordination exhibited by PsaA may have a physiological role in aiding ligand release [40]. Further structural and functional studies will be required to better understand the impact of these subtle, intriguing and potentially important differences in metal coordination schemes between the SBPs present in many different bacterial species.

In SitA, the Mn^2+^ ion is bound in a polar and electrostatic environment, with hexavalent octahedral coordination via the side chains of His^64^, His^137^, Glu^203^ and Asp^278^ (Figure 4). This direct protein–metal interaction is in contrast with the indirect capture of Fe^3+^ by siderophore-binding SBPs, as exemplified by the Fe^3+^–ferrichrome–FhuD2 interaction [19]. Here, in our crystallographic ‘snapshot’, the Mn^2+^ ion is engulfed and completely buried from the solvent (Figure 6B, upper panel). Presumably, binding of the Mn^2+^-bound SitA lipoprotein to its membrane-bound receptor, SitBC, can promote conformational changes in SitA sufficient to induce release of its metal cargo, in order to enable nutrient uptake and recycling of the apo-form of the transport protein. We wished to explore this hypothesis by studying interactions of apo- and ligand-bound forms of SitA with recombinant SitB or SitBC. However, despite extensive efforts, we were unable to produce a soluble form of the integral membrane protein SitB alone or in complex with SitC.

Subsequently, we also determined the structure of SitA bound to Zn^2+^ (Figure 4). The two metal-bound SitA structures were highly similar overall, although in particular the position of the ion-chelating His^64^ side chain was different. The possibility to undergo these minor local rearrangements, hosted upon an unchanging scaffold presenting four tetrahedrally arranged metal-chelating amino acids, may underlie the observed ability of SitA to bind to several different divalent metal cations. Comparison of the Zn^2+^-bound SitA structure with another zinc-bound SBP, the *E. coli* protein ZnuA, revealed considerable fold similarity (rmsd 2.2 Å) but to a lesser extent than when comparing SitA with the Mn^2+^-binding protein PsaA (see above and Supplementary Figure S5). A characteristic feature that may distinguish the Zn^2+^-binding SBP is in the cation-binding site residues and their organization. Typically, the Mn^2+^ binding site is composed by two histidines, one glutamate and one aspartate, whereas the Zn^2+^-binding site instead typically involves three histidines and one aspartate or glutamate (Supplementary Figure S5) [41–43]. Interestingly, while these SBPs display subtle variations on the same structural motif to determine distinct binding specificities, it has also been hypothesized that the streptococcal Lbp (laminin-binding protein) has undergone convergent evolution to enable Zn^2+^-coordination via a metal-binding site with a geometry similar to those of several SBPs including ZnuA [54].

Measurements by ITC revealed that SitA binds very tightly to both Mn^2+^ and Zn^2+^ ions, although the estimated values (low nanomolar range *K*_D_) were close to the limits of the technique [52] and should therefore be considered as apparent affinity estimates. Mutations to alanine in the chelating residues strongly reduced the metal-induced stabilization observed in DSC; and, moreover, the double mutant E203A+D278A was sufficient to completely abolish binding to Zn^2+^ or Mn^2+^ in ITC experiments (Figure 7). Overall, these insights coupled with the availability of characterized non-binding mutants of SitA provide a platform for future investigations of the physiological role of SitA during colonization and/or invasion in animal models of disease caused by *S. pseudintermedius*. The observations discussed suggest that *S. pseudintermedius* may use the highly conserved SitA metal-binding residues to capture and transport a range of nutrients, likely with a preference for Mn^2+^ ions for which it displayed the canonical commonly observed geometry and bond lengths [49,50]. Nonetheless, the structure of MtsA bound to Fe^2+^ [23] showed how the same four conserved and spatially equivalent chelating residues in a different SBP protein can be used to bind yet another different divalent metal cation (iron). Since the overall structures and, more importantly, the spatial arrangement of all four metal-binding residues, are conserved in homologous proteins from other species (Figure 5), it seems likely that SitC in *S. epidermidis*, MntC in *S. aureus*, PsaA in *S. pneumoniae*, and MtsA in *S. pyogenes*, may also display a degree of promiscuity in ligand binding. Which metals are actually bound and efficiently transported *in vivo* will likely also depend on metal bioavailability in the niche being colonized. This issue has been studied and discussed to some extent for PsaA, where it was concluded that a greater stability of the Zn^2+^-bound form, compared with the Mn^2+^ bound form, of PsaA might represent a mechanism for the antibacterial effect of Zn^2+^ [24]. Whether this mechanistic hypothesis holds for the SBPs of other species remains to be determined.

Finally, in addition to the Mn^2+^ and Zn^2+^ bound structures of SitA, we also obtained a ligand-free, apo-form of SitA. Notably, a search of the PDB using the protein structure comparison service, Fold [55], revealed 12 different (non-redundant) SBPs for which over 60 distinct polypeptide chain structures have been determined and which can all be pairwise-aligned with SitA with rmsds <3.0 Å. All such structures are SBPs with divalent metal-binding properties. The majority of these distinct protein structures (at least eight, to date) have been determined only in the metal-bound state. None of these protein structures have been determined only in the apo-form, and only three of these structures have been determined as wild-type proteins both in the metal-bound and metal-free states, potentially allowing mechanistic insights. However, in the apo-form of TroA from *T. pallidum*, despite the absence of a metal ion, the protein displays a closed state, with all potential metal-binding residues (His^68^, His^133^, His^199^, Asp^279^) showing inward-facing positions [56]. Similarly, inspection of apo- and holo-forms of ZnuA from *E. coli* reveals that both show a closed state with all potential metal-binding residues (Glu^59^, His^60^, His^143^, His^207^) in inward-facing positions compatible with metal binding [57]. Interestingly, the most recent related study provided the apo-form structure of wild-type PsaA from *S. pneumoniae* (PDB 3ZK7) [40], revealing an outward-facing histidine analogous to His^64^ in SitA (Figure 6A). As such, PsaA and the SitA structures determined herein offer a rare possibility to visually assess the detailed structural and conformational effects of ligand binding in an SBP.

The comparison of apo- and holo-forms of SitA shows how the binding of a metal ion coincides with changes in the structures of at least two neighbouring loops. Interestingly, in the SitA apo-form, the side chain of His^64^ is flipped outwards, adopting a position incompatible with the full canonical metal coordination scheme. It is conceivable that with these two loops and His^64^ in the outward ‘open’ position, Mn^2+^ can readily enter the binding pocket of the SitA apo-form via the open tunnel (Figure 6B, lower panel). After metal binding, the large reorientation of His^64^ into the inward ‘closed’ position, and clamping-down of the two neighbouring loops, effectively close the tunnel (Figure 6B, upper panel), resulting in high-affinity ligand binding. Similarly, ligand-dependent rearrangement of the corresponding residue His^60^ in the Zn^2+^-binding site of *E. coli* ZnuA has been reported [57]. Collectively, these findings suggest that this His residue, located at the outer edge of the binding pocket and very highly conserved in metal-binding SBPs, may mediate a conserved pivotal role in ion binding and release.

These structural rearrangements may be the mechanism triggering the ligand-dependent engagement of the SitBC receptor required for nutrient metal uptake and essential for bacterial virulence. On the basis of sequence and structure conservations, a similar binding and transport mechanism may be conserved in numerous SitA protein homologues. In summary, our results provide the first experimentally determined mechanistic and functional insights into this surface-exposed lipoprotein from *S. pseudintermedius*, a bacterial pathogen of animals and humans, of growing veterinary and medical importance.

## Online data

Supplementary data
