# Supplementary material for: Apo, Zn2+-bound and Mn2+-bound structures reveal ligand-binding properties of SitA from the pathogen Staphylococcus pseudintermedius
Source: Biosci Rep. 2014 Nov 24;34(6):e00154. doi: 10.1042/BSR20140088 (PMC4242081; doi:10.1042/BSR20140088)
Supplement: Supplementary data [file bsr034e154ntsadd.pdf]

SUPPLEMENTARY FIGURE S1 (ABATE F ET AL)

A

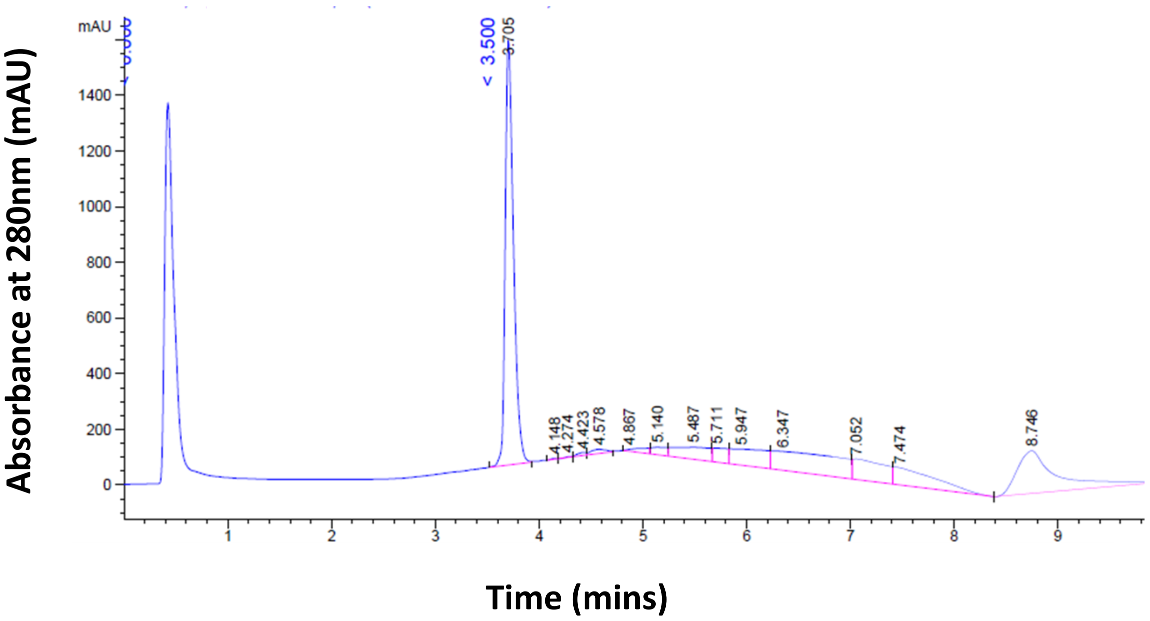

B

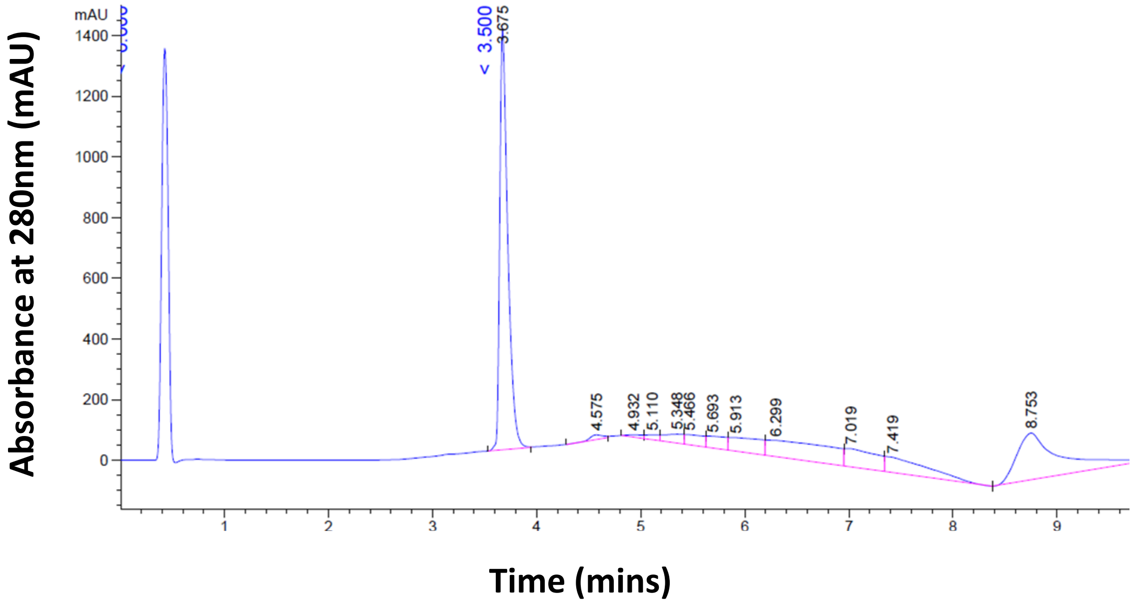

Legend S1 The two panels show the elution profiles of reverse-phase high-performance liquid chromatography (RP-HPLC) analyses of wild type SitA (panel A, >98 % purity) and SitA double-mutant E203A + D278A (panel B, >97% purity). Both proteins elute after approximately 3.7 minutes.

SUPPLEMENTARY FIGURE S2 (ABATE F ET AL)

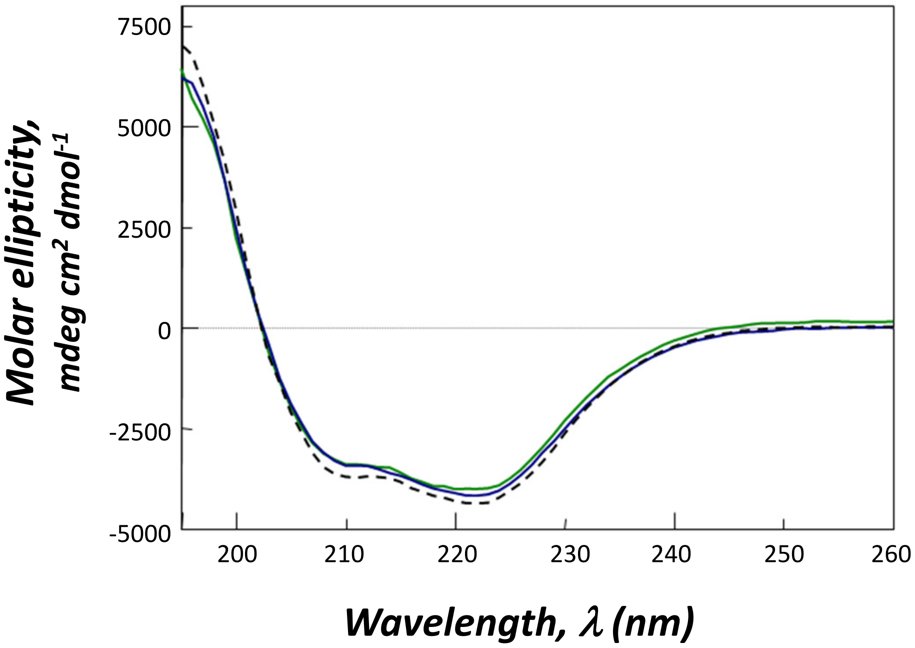

Legend S2 Far-UV CD spectra of SitA apo-form (black, dashed line), and SitA in presence of 2mM $MnCl_2$ (blue line) or 2mM $ZnCl_2$ (green line). There are no major differences observed under the different conditions. All spectra display the double minima at approximately 208nm and 222nm, typical of the presence of α-helical secondary structure elements.

SUPPLEMENTARY FIGURE S3 (ABATE F ET AL)

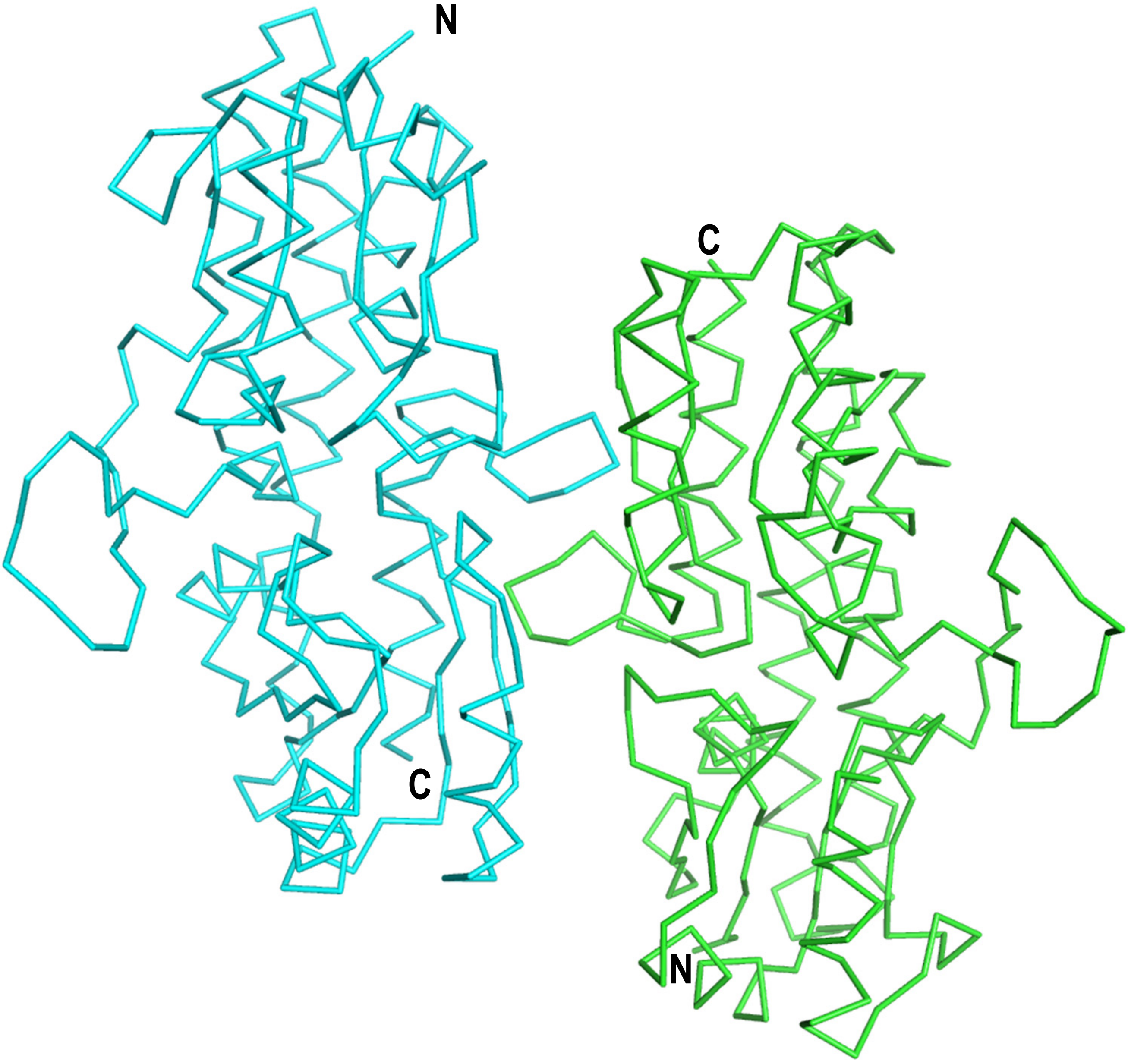

Legend S3 The protein crystals obtained in the presence of Mn^{2+} contained two SitA chains per asymmetric unit, arranged in a 'face-to-face' manner. Here, the two chains are shown in 'ribbon' representation; N and C termini are labelled. The figure was prepread using Pymol.

SUPPLEMENTARY FIGURE S4 (ABATE F ET AL)

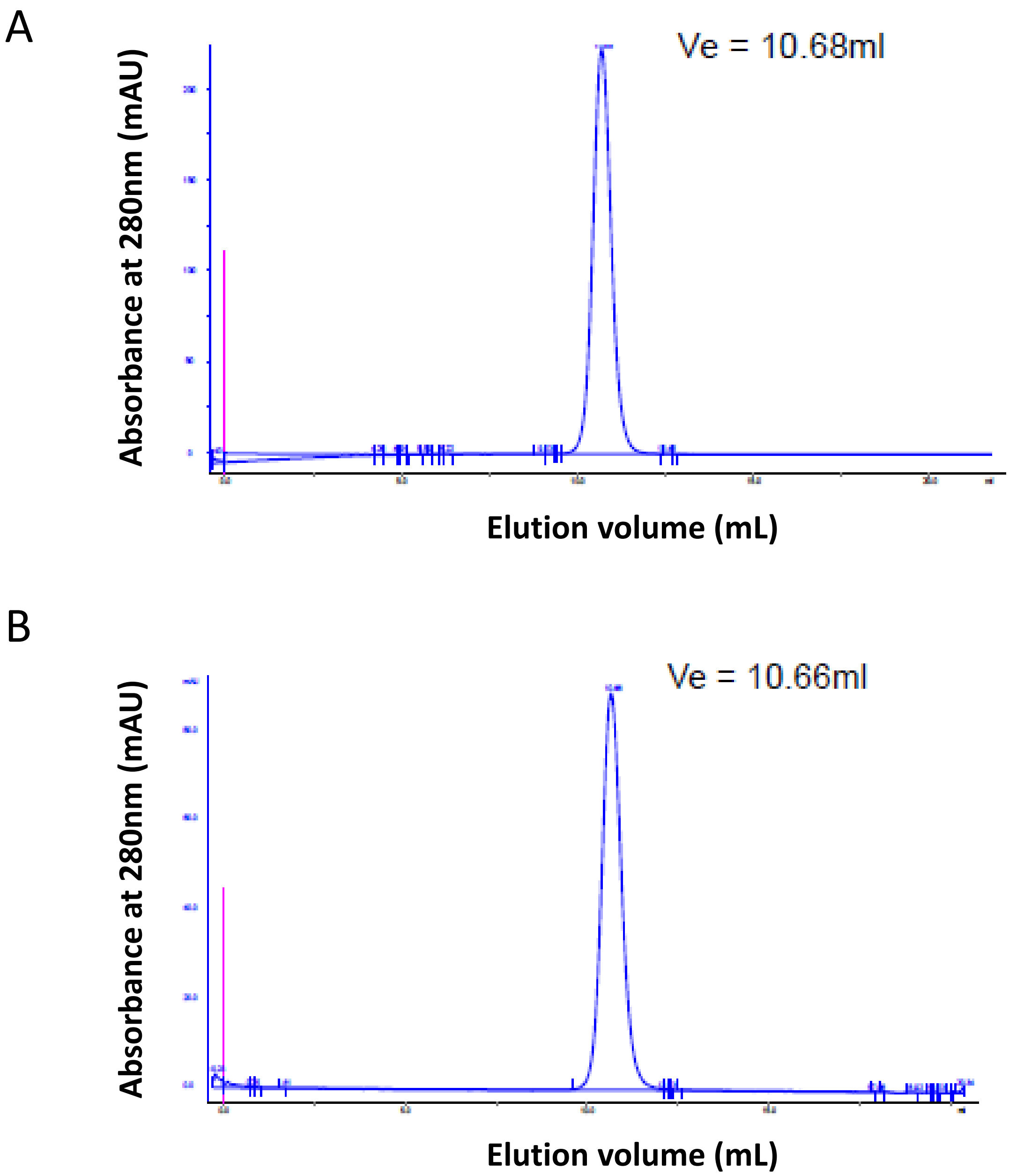

Legend S4 The two panels show the elution profiles of analytical size-exclusion chromatography (SEC), as a plot of UV-Absorbance units at 280nm (mAU) against elution volume (mL); the elution volume at maximal absorbance is indicated (Ve). Analytical SEC was used to assess the apparent molecular weight of the recombinant wild type SitA protein alone (panel A) or in the presence of 10mM divalent metal cations (panel B; here the elution profile is shown for SitA + 10mM $ZnCl_2$; similar results were obtained in presence of $MnCl_2$). The protein elutes as a monomer and the profile is not changed by the presence of divalent metal cations.

SUPPLEMENTARY FIGURE S5 (ABATE F ET AL)

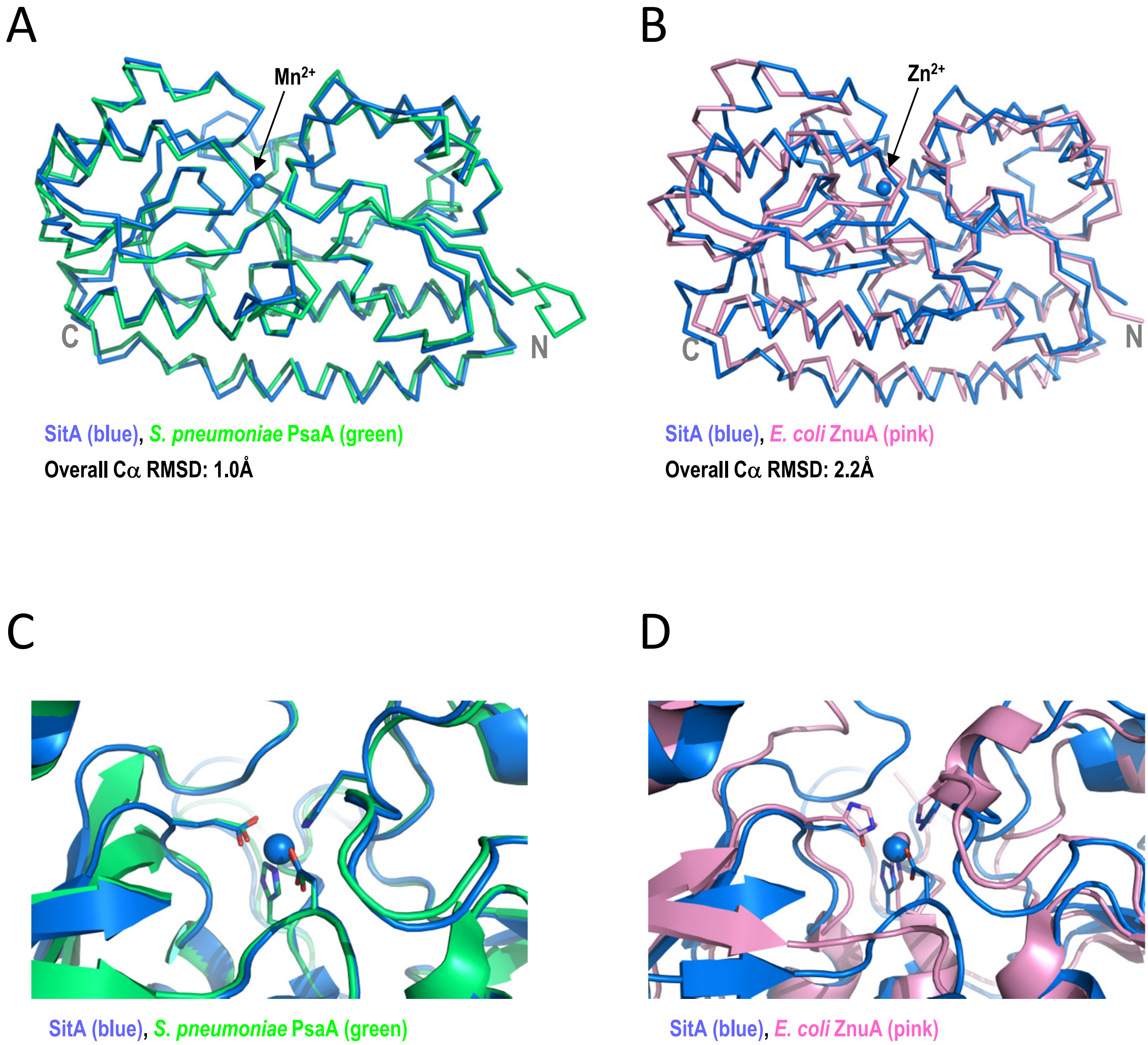

Legend S5 Ribbon plots for SitA (blue) superimposed onto PsaA (green, panel A) and ZnuA (pink, panel B). The overall folds of all three molecules are very similar, including the size and location of their metal ion-binding pockets. There is greater structural similarity of SitA with PsaA, as reflected in the lower rmsd score.

Panels C and D: zoomed images of the binding pockets from panels A and B. The panel C shows the preferred Mn-binding scheme of His, His, Glu, Asp; while panel D shows the preferred Zn-binding scheme of His, His, His, Asp, which indeed shows greater differences with the SitA structure. All figures were prepared using Pymol.
